# δ/ω-Plectoxin-Pt1a: An Excitatory Spider Toxin with Actions on both Ca^2+^ and Na^+^ Channels

**DOI:** 10.1371/journal.pone.0064324

**Published:** 2013-05-14

**Authors:** Yi Zhou, Mingli Zhao, Gregg B. Fields, Chun-Fang Wu, W. Dale Branton

**Affiliations:** 1 Department of Biomedical Sciences, Florida State University College of Medicine, Tallahassee, Florida, United States of America; 2 Department of Biology, University of Iowa, Iowa City, Iowa, United States of America; 3 Torrey Pines Institute for Molecular Studies, Port Saint Lucie, Florida, United States of America; 4 Department of Neuroscience, University of Minnesota Medical School, Minneapolis, Minnesota, United States of America; Yale School of Medicine, United States of America

## Abstract

The venom of spider *Plectreurys tristis* contains a variety of peptide toxins that selectively target neuronal ion channels. O-palmitoylation of a threonine or serine residue, along with a characteristic and highly constrained disulfide bond structure, are hallmarks of a family of toxins found in this venom. Here, we report the isolation and characterization of a new toxin, δ/ω-plectoxin-Pt1a, from this spider venom. It is a 40 amino acid peptide containing an O-palmitoylated Ser-39. Analysis of δ/ω-plectoxin-Pt1a cDNA reveals a small precursor containing a secretion signal sequence, a 14 amino acid N-terminal propeptide, and a C-terminal amidation signal. The biological activity of δ/ω-plectoxin-Pt1a is also unique. It preferentially blocks a subset of Ca^2+^ channels that is apparently not required for neurotransmitter release; decreases threshold for Na^+^ channel activation; and slows Na^+^ channel inactivation. As δ/ω-plectoxin-Pt1a enhances synaptic transmission by prolonging presynaptic release of neurotransmitter, its effects on Na^+^ and Ca^2+^ channels may act synergistically to sustain the terminal excitability.

## Introduction

Spider venoms are a rich source of biological neurotoxins that affect synaptic transmission [1]–[4]. From the venom of the spider *Plectreurys tristis*, we and others have identified several toxins that are potent inhibitors of synaptic transmission in insects [5]–[8]. The toxins that we have characterized were originally designated PLTX with a Roman numeral suffix that relates to a specific toxin peak in reverse-phase HPLC. PLTX II (or ω-PLTX-Pt1a in the new nomenclature system) rapidly and irreversibly inhibits synaptic transmission at *Drosophila* neuromuscular junctions by selectively blocking presynaptic Ca^2+^ channels [9]. PLTX II is a 44 amino acid peptide with an O-palmitoyl threonine amide at its carboxyl terminus. We have shown that the lipid component is required for the biological activity of PLTX II, suggesting that fatty acylation plays an important role in a key aspect of the action of the toxin [10], [11].

Lipid modification of proteins, including myristoylation, prenylation and palmitoylation, is a universal phenomenon and may serve to tether the fatty acylated proteins to the plasma membrane or act through other molecular mechanisms [12]–[15]. PLTX II was the first example of O-linked palmitoylation for a biologically active peptide. The underlying biochemistry of O-palmitoylation is likely different from that of most previously characterized palmitoylation of proteins, in which palmitic acid is linked to cysteine residues by thioesterification (S-palmitoylation) [16]. The O-palmitoyl linkage is much more stable than the S-palmitoyl linkage and may be best suited for permanent modification of proteins as opposed to the S-palmitoylation found in highly reversible regulatory processes. It is also conceivable that S-palmitoylation might be stabilized through conversion to O-palmitoylation in some instances. *Plectreurys* venom contains toxins with variety of biological activities [5], [7], [17]. Most of these toxins have an apparent MW range of 4-7 kDa, and many elute close to PLTX II on C18 RP-HPLC in a region where relatively hydrophobic peptides of this size would be expected to elute. When this group of apparently hydrophobic peptides is treated with base, the result is a large hydrophilic shift of much of the material on RP-HPLC, associated with a loss of biological activity. This suggests that fatty acylation is a common modification of peptide toxins in *Plectreurys* venom. Quistad and Skinner reported amino acid sequences of several potent insecticidal *Plectreurys* toxins derived from the same general region in RP-HPLC [7]. Although they did not characterize any lipid modifications analogous to the palmitoylation we had previously shown for PLTX II, they did acknowledge the possibility that a C-terminal modification might be present. Toxins characterized in their studies are similar in size and primary structure to the toxins we have characterized. Amino acid sequences are hydrophilic but the mature toxins are strongly retained in RP-HPLC [7], [10]. Thus, it is highly probable that they are also fatty acylated.

We have now fully characterized a new *Plectreurys* toxin with novel biological activity. The toxin, designated δ/ω-plectoxin-Pt1a (δ/ω-PLTX-Pt1a) according to the rational nomenclature system [18], has an O-palmitoyl modification at a near C-terminal serine residue. Consistent with our previous findings of PLTX II, δ/ω-PLTX-Pt1a appears to block a specific subset of neuronal Ca^2+^ channels in *Drosophila*, as indicated by Ca^2+^ imaging of cultured neurons. Remarkably, however, δ/ω-PLTX-Pt1a has an excitatory effect at *Drosophila* neuromuscular junction, manifested as prolonged release of neurotransmitter from presynaptic terminals. Direct patch-clamp measurements on *Drosophila* neurons demonstrate that δ/ω-PLTX-Pt1a alters both Ca^2+^ and Na^+^ channels. In addition to a partial blockade of Ca^2+^ influx, the toxin shifts the activation voltage and slows the inactivation process of Na^+^ channels rendering the axonal terminal hyperexcitable. This unique activity suggests that δ/ω-PLTX-Pt1a may be useful in identifying Ca^2+^ channels that are specifically involved in control of nerve terminal excitability and in revealing the common molecular domains in Na^+^ and Ca^2+^ channels that are susceptible to modifications by δ/ω-PLTX-Pt1a. The relatively small size, shared structural motifs, and limited precursor structure of this family of toxins may also provide a model for studies of the biochemistry of O-palmitoylation.

## Materials and Methods

### Reagents

The crude venom of spider *Plectreurys tristis* was purchased from Spider Pharm, Feasterville, PA. Trypsin was obtained from Promega. Tetrodotoxin (TTX) was purchased from Calbiochem. Trifluoroacetic acid (TFA) and heptafluorobutyric acid (HFBA) were sequanal reagents from Pierce. Water and acetonitrile (ACN) were HPLC grade.

### Purification of δ/ω-PLTX-Pt1a

δ/ω-PLTX-Pt1a was purified from crude venoms by size exclusion and two steps of reverse-phase HPLC (RP-HPLC) as previously described for purification of PLTX II [5]. Briefly, 500 µl crude venom was diluted 1:1 with aqueous 0.1% TFA and fractionated by size exclusion on a Sephadex G50 column at a flow rate of 2.5 ml/min. The G50 fractions were loaded onto a semi-preparatory C18 column (Vydac 218TP510) and eluted in 0.1% TFA with an ACN gradient from 0 to 60% at a flow rate of 4.7 ml/min. δ/ω-PLTX-Pt1a was finally purified on an analytical C18 column (Vydac 218TP54) in 0.1% HFBA with an ACN gradient from 45 to 48% at a flow rate of 1.0 ml/min.

### Trypsin digestion and peptide sequencing

Purified δ/ω-PLTX-Pt1a (7–10 nmole) was dissolved in 200 µl of 8 M Urea, 0.1 mg/ml EDTA, 5 mg/ml DTT and 50 mM Tris-HCl (pH 7.6), and incubated under argon at 20°C for 3 h. After adding 5 µl of 4-vinylpyridine, tubes were incubated at 20°C for an additional 1.5 h. The reduced and pyridethylated δ/ω-PLTX-Pt1a was acidified with 10 µl TFA and purified by RP-HPLC on an analytic C18 column (Vydac 218TP54) with a linear gradient from 0–60% ACN in 0.1% TFA at a flow rate of 1.0 ml/min. After purification, the reduced and pyridethylated δ/ω-PLTX-Pt1a was digested by trypsin (1 µg/50 µl) in reaction buffer (50 mM NH_4_HCO_3_, pH 7.8) at 37°C for 3 h. Tryptic fragments of δ/ω-PLTX-Pt1a were then separated on an analytical RP-HPLC C18 column using and ACN gradient with 0.1% TFA as described above.

The reduced and pyridethylated δ/ω-PLTX-Pt1a or tryptic fragments were covalently attached to a Milligen arylamine sequencing membrane with carbodiimide using a Milligen kit (Bedford, Mass). They were sequenced on the membrane in an ABI 477A sequencer following standard ABI protocols.

### Cloning of δ/ω-PLTX-Pt1a cDNA

Cloning of δ/ω-PLTX-Pt1a cDNA was accomplished in a straightforward manner using a sequential series of PCR amplifications with a *Plectreurys* cDNA library provided by Sandoz Agro. cDNAs were synthesized and cloned in the λZAPII system starting with *P. tristis* cephalothorax mRNAs [17]. Initially, degenerate PCR primers were designed based on two parts of the peptide sequence and these primers were used in PCR with vector-specific primers, using amplified aliquots of the library as template. A forward primer was based on the sequence CAGWN, near the N-terminal of the known sequence, and a reverse primer was designed at CTGTK, near the C-terminal. Each of these was used in PCR with T3 and T7 vector sequencing primers to produce PCR products that included either end of the toxin cDNA insert. PCR products were isolated from agarose gel electrophoresis, cloned into a pCR2.1-TOPO vector and multiple clones were sequenced to find clones with sequence matching amino acid sequence of the peptide. Perfect forward and reverse primers derived from the ends of these sequences were used in PCR to clone the complete toxin cDNA.

### Mass Spectrometry analyses

Masses of native, reduced and pyridethylated deacylated δ/ω-PLTX-Pt1a and all the tryptic fragments were measured by mass spectrometry. Approximately 1 nmol of each sample was dissolved in 100 µl of a solvent consisting of 80% ACN and 1% formic acid. Solution was then introduced into a Sciex API III tandem mass spectrometer at a flow rate of 5 µl/min via an ion-spray interface.

### Electrophysiology at neuromuscular junctions

Standard electrophysiological techniques were used to record from the *Drosophila* neuromuscular junction [19]. Briefly, a late third-instar larva was cut along the dorsal middle line, pinned out and eviscerated. The nerves innervating the body wall muscles in the abdominal segments were cut at the ganglion end and stimulated with a suction electrode. The nerve was stimulated once every 5 sec with a 0.2 msec pulse at twice the threshold for activation. The excitatory junction potentials (EJPs) were recorded from one of the ventral longitudinal muscles using a 3 M KCl-filled glass microelectrode (∼20 MΩ). The physiological saline contained (in mM) 120 NaCl, 2 KCl, 4 MgCl_2_, 0.6 CaCl_2_, 36 sucrose, and buffered at pH 7.3 with 5 mM HEPES. Extracellular recordings of nerve action potential were made from a small loop of motor nerve drawn into a second tight-fitting glass suction electrode.

### Cell culture

Cultures of *Drosophila* “Giant” neurons, derived from cytokinesis-arrested embryonic neuroblasts, were prepared as described previously [20]. Briefly, Embryos were collected on agar plates (1–2 h) and incubated for 3.5 h at 25°C. After homogenizing embryos in modified Schneider medium supplemented with 20% fetal bovine serum (FBS), insulin (200 ng/ml), penicillin (50 U/ml), and streptomycin (50 U/ml), cells were dissociated and then plated on glass coverslips in the above medium containing l–2 µg cytochalasin B (Sigma). Cultures were maintained in humidified chambers at room temperature (22–23°C).

### Whole-cell recording

Patch electrodes with tip resistance of 3–6 MΩ were made from glass capillaries, and coated with Sylgard to reduce stray capacitance. Whole-cell recordings were obtained primarily from monopolar or bipolar neurons approximately 10 µm in soma diameter using an EPC-7 or Axopatch 2B amplifier. Standard bath solution contained (in mM) 128 NaCl, 2 KCl, 4 MgCl_2_, 1.8 CaCl_2_, and 35.5 sucrose, buffered with 5 mM HEPES at pH 7.1–7.2. For inward current measurement, K^+^-free (replaced by Cs^+^) pipette solution was used to reduce outward K^+^ current contamination as previously described [21], [22]. It contained (in mM) 144 CsCl, 1.0 MgCl_2_, 0.5 Ca_2_CI, and 5 EGTA, buffered with 10 mM HEPES, pH 7.1–7.2. To isolate voltage-activated Ca^2+^ currents, 0.25 µM TTX was added to the bath solution to block Na^+^ currents. Na^+^ currents were isolated by blocking Ca^2+^ channels with Cd^2+^. To assess steady-state inactivation of Na^+^ currents, the test pulse (−10 mV) was delivered after 500 ms preconditioning pulses varying from −100 to +60 mV at 10 mV increments. From which, the membrane potential of half-inactivation (V_h_) and the fractional recovery (r) were determined. Time constant of inactivating Na^+^ currents (τ) was measured by single exponential fitting of the current decay phase evoked by depolarizing pulse to −20 mV from the holding potential of −80 mV. In few cases, currents were better fitted with a double exponential function.

### Calcium imaging

Primary neuronal cultures were prepared from *Drosophila* embryos according to standard methods. Cells were plated on 96 well culture plates, which were constructed so that a glass was directly attached to the bottom of the culture plate. Cells were grown in modified Schneider's medium supplemented with 20% fetal calf serum and insulin in an incubator at 22°C.

After at least 24 h, cells were loaded with Ca^2+^ indicator dye Fura-2 by incubation for 30 min at room temperature in plating medium containing 5 µM Fura-2 AM. Following loading, cells were washed briefly with *Drosophila* saline which was composed of (in mM) 5 HEPES, 120 NaCl, 2 KCl, 2 CaCl_2_, 4 MgCl_2_, and 36 sucrose (pH 7.3). One of the wells was viewed with a 40× objective on an inverted fluorescence microscope equipped with an intensified CCD camera and commercial image acquisition hardware and software (Quantex). Cells were illuminated alternatively with 340 and 380 nm light from a PTI monochromator, and the emission of the dye was monitored at λ = 510 nm. Images were stored and ratioed after background subtraction. The ratioed images were calibrated using standard calcium solutions to represent intracellular calcium concentrations in the imaged cells.

Ca^2+^ influx was induced by a brief application of a saline in which potassium concentration was increased to 80 mM by substitution of potassium for sodium. High potassium solution (80 mM) and toxins were administered via a 0.5 ml injection loop.

### Statistical analysis

Statistical analyses were performed with the GraphPad Prism software using student's *t*-tests. *p*<0.05 was considered statistically significant.

## Results

### Purification of δ/ω-PLTX-Pt1a

**Figure 1:**
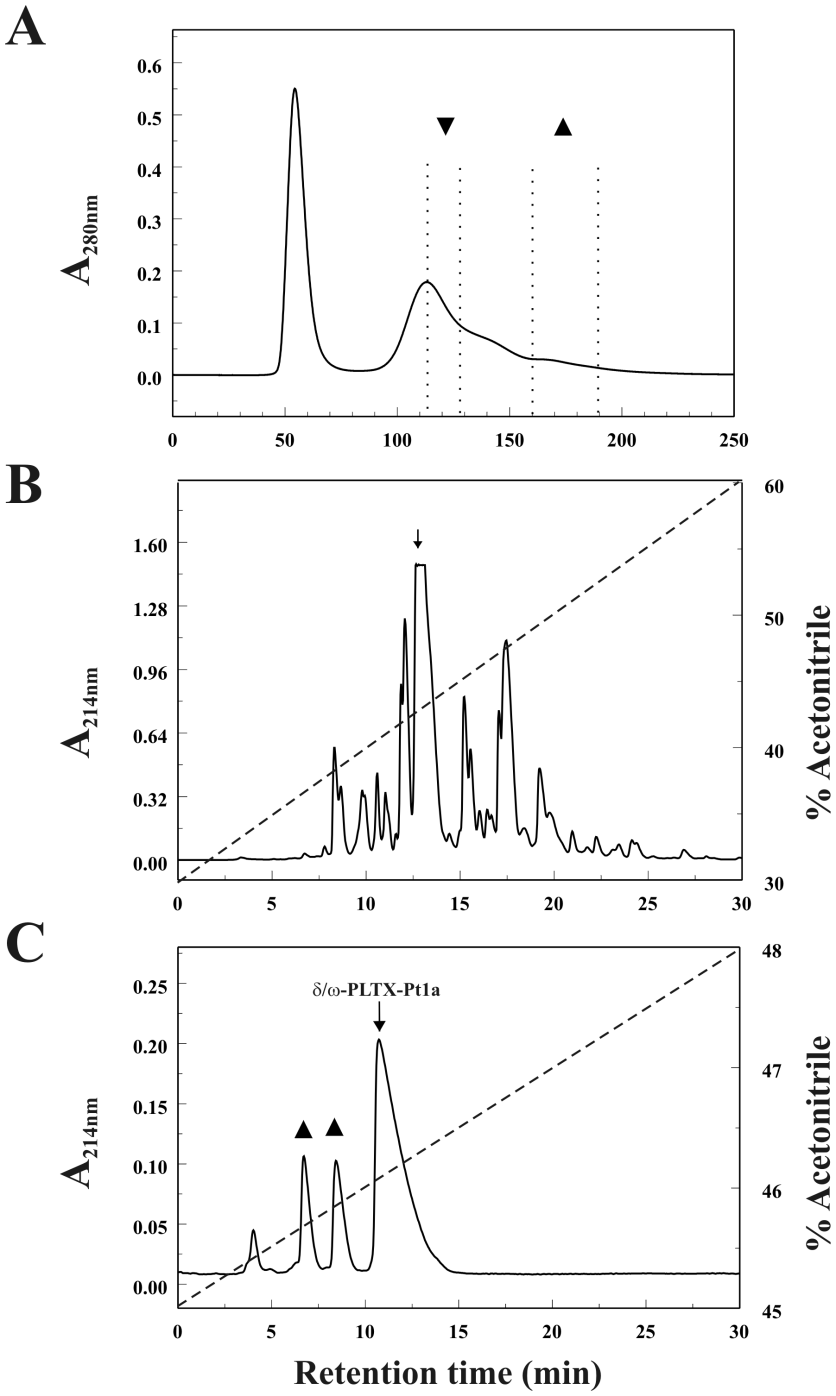
Purification of δ/ω-PLTX-Pt1a. **A**, Crude *Plectreurys* venom was fractionated on a HPLC G-50 column. Further purification was done with the fraction (▴) taken from the later part of the second complex peak. Insect-specific inhibitory toxins including PLTX II were previously purified from another G50 fraction (▾). **B**, The G-50 fraction was separated by RP-HPLC on a C18 column and eluted with a gradient of 30% to 60% ACN in 0.1% TFA, as shown by the dashed line. **C**, The predominantly active fraction (indicated by the arrow in **B**) was further purified on an analytic C18 column in a second solvent system containing 0.1% HFBA, which separates δ/ω-PLTX-Pt1a (arrow) from two inhibitory peaks (▴).

G-50 size exclusion separates crude *Plectreurys* venom into two major peaks (Fig. 1A). The first peak contains components with relatively high molecular weight including proteases. All the peptide toxins that affect synaptic transmission are in a second complex peak [5]. In this study, a G50 fraction taken from a later portion of the complex peak was chosen for further purification (Fig. 1A). This fraction was re-fractionated by RP-HPLC on a Vydac C18 column (Fig. 1B). Several peaks were found to be active in blocking synaptic transmission at *Drosophila* neuromuscular junction. The predominantly active fraction was then further separated on a Vydac analytic C18 column in a second solvent system containing 0.1% HFBA. As shown in Figure 1C, four homogeneous peaks were revealed. We assayed these peaks in *Drosophila* neuromuscular junction preparation, and found that two of them inhibited synaptic transmission (Fig. 1C). The fourth peak was the most abundant one. It had very little effect on the amplitude of the excitatory junction potential (EJP), but instead prolonged the EJP evoked by nerve stimulation. Mass spectrometry suggested that this peak contained a homogeneous toxin with a mass of 4646 AMU. We named this toxin δ/ω-PLTX-Pt1a based on its molecular targets (see below).

### Structural determination

**Figure 2:**
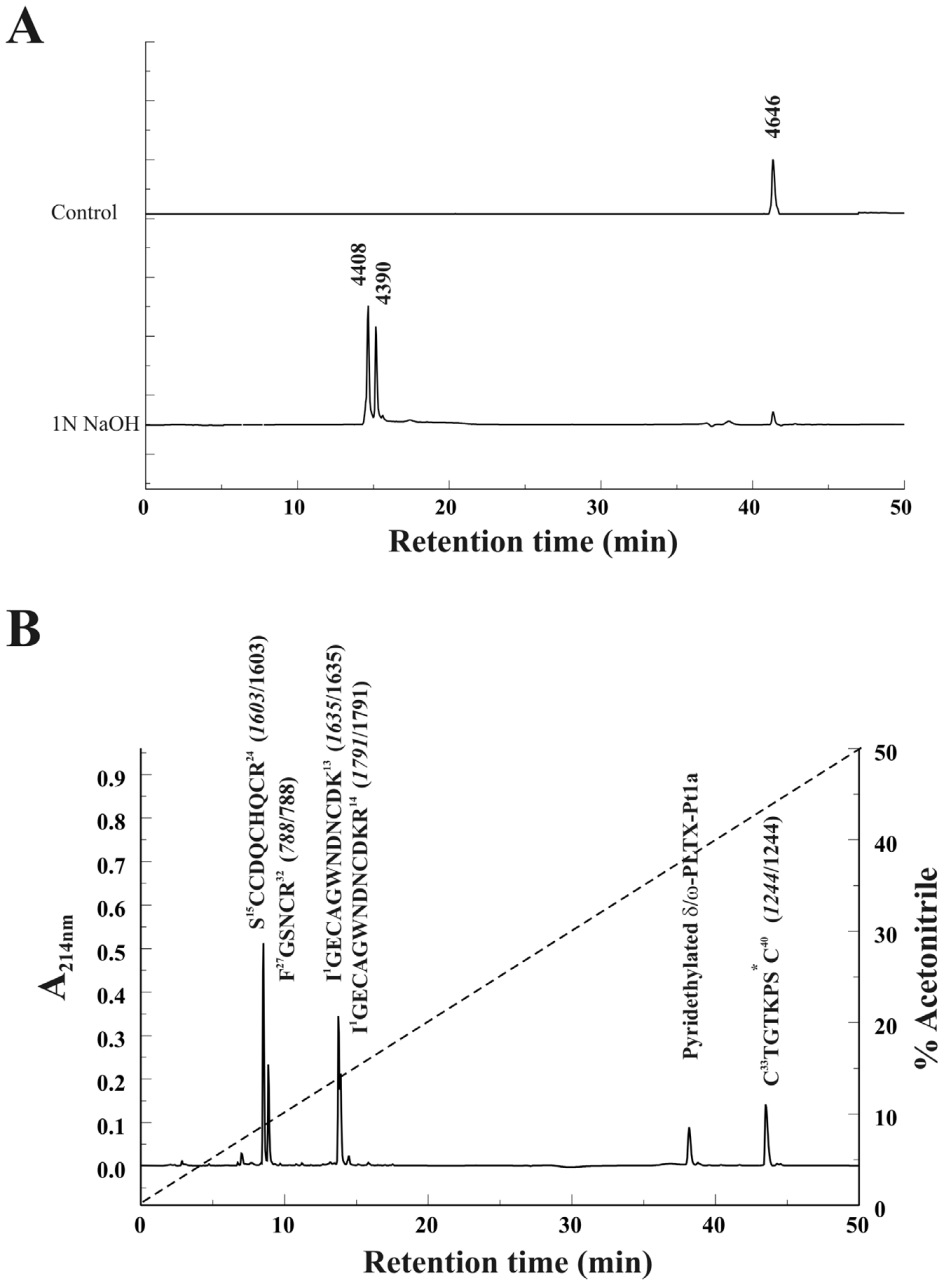
Structural characterization of δ/ω-PLTX-Pt1a. **A**. Native δ/ω-PLTX-Pt1a was subjected to alkaline depalmitoylation with 1N NaOH (pH 10.0) for 60 min, and analyzed by RP-HPLC with a gradient of 0–60% ACN in 0.1% TFA. **B**. Fragments generated from trypsin digestion of reduced and pyridethylated δ/ω-PLTX-Pt1a were separated on a C18 RP-HPLC column. Amino acid sequences for these fragments are given above each peak, followed by the masses predicted from their sequences (italic fonts) and the masses measured by mass spectrometry. The asterisks (*) indicate fatty modification at the C-terminal fragment.

The size and RP-HPLC retention time of δ/ω-PLTX-Pt1a were consistent with a fatty acylated peptide structure similar to that of PLTX II. Treatment of the native δ/ω-PLTX-Pt1a with 1 N NaOH to cleave this putative ester resulted in two products. As shown in Figure 2A, both products underwent a significant hydrophilic shift relative to the native toxin and eluted very close to each other on RP-HPLC. The earlier of these two peaks was 238 Da smaller than the native toxin, consistent with loss of a palmitoyl moiety with regeneration of a hydroxyl group in the peptide. An analogous product is produced when the O-palmitoyl Thr ester in PLTX II is cleaved by the same treatment [10], [11]. The second peak, an analog of which was not found with base treatment of PLTX II, was 256 DA smaller than the native toxin. This is the shift in mass equivalent to loss of the palmitoyl moiety concomitant with loss of an additional H_2_O from the peptide. This is likely due to base-catalyzed, β elimination-dehydration of O-modified Ser residues to dehydroalanine [23], [24]. Modified Ser residues are much more susceptible to this reaction than are modified Thr residues [25], [26]. The abundance of the dehydration product in base-treated material was a preliminary indication that δ/ω-PLTX-Pt1a might be palmitoylated at a Ser residue rather than at a Thr as we had previously found in PLTX II.

**Figure 3:**
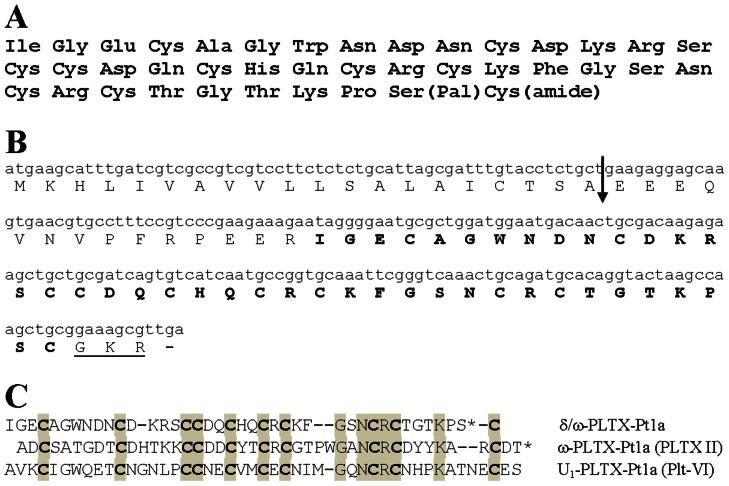
δ/ω-PLTX-Pt1a sequences. **A**. Complete amino acid sequence of δ/ω-PLTX-Pt1a. Pal: O-palmitoyl moiety; amide: amidated C-terminal. **B**. cDNA coding sequence and amino acid sequence of δ/ω-PLTX-Pt1a. The mature toxin is indicated by bold fonts, and the amidation signal sequence at the C-terminus is underlined. The arrow denotes a putative signal cleavage site [17], [27]. **C**. Comparison of amino acid sequences of δ/ω-PLTX-Pt1a, ω-PLTX-Pt1a (PLTX II) and U_1_-PLTX-Pt1a (Plt-VI). Plt-VI was reported by Quistad and Skinner [7]. Identical residues in all three peptides are shown in *shaded boxes*. Spacers (-) are inserted to show maximal homology. The asterisks (*) denote O-palmitoylated residues.

Edman microsequencing of reduced and pyridethylated δ/ω-PLTX-Pt1a allowed the assignment of amino acid residues 1–38 (Fig. 3A). C-terminal fragments underwent unusually severe washout during each cycle, a phenomenon also observed with PLTX II as a result of the fatty acylation. This made it essentially impossible for us to determine the C-terminal sequence by microsequencing alone. We thus carried out additional experiments to determine the complete structure using approaches similar to those utilized in the characterization of PLTX II structure [10], combined with cloning of cDNA coding for the toxin precursor.

Mass spectrometry (MS) analysis of the reduced and pyridethylated δ/ω-PLTX-Pt1a yielded a mass of 5708 Da, which was larger than the native toxin by an amount equivalent to the pyridethylation of 10 half-cystines, indicating that δ/ω-PLTX-Pt1a contains 10 Cys residues in disulfide bonds. Since only 9 Cys residues were identified among the 38 amino acids determined by microsequencing, we could infer that one of the unidentified residues was Cys and the remaining mass of the toxin could be approximately accounted for by a palmitoylated Ser residue.

A sample of the reduced and pyridethylated δ/ω-PLTX-Pt1a was fragmented by trypsin digestion. As shown in Figure 2B, five fragments and one small peak containing undigested δ/ω-PLTX-Pt1a were separated by RP-HPLC. Four peaks, representing non-acylated fragments, eluted early. MS analysis of these fragments yielded masses of 1603, 788, 1635 and 1791 Da. These values matched the calculated masses of four predicted tryptic fragments derived from N-terminal amino acids 1–33 as determined by microsequencing (Fig. 2B). The sixth fragment eluted later than the undigested δ/ω-PLTX-Pt1a and likely contained the fatty acylated C-terminal region (Fig. 2B). Analysis of predicted trypsin cleavage sites suggested that this was a C-terminal fragment beginning with Cys_33_ (due to cleavage after Arg_32_), and the partial sequence of this fragment obtained from microsequencing was Cys_33_-Thr_34_-Gly_35_-Thr_36_-Lys_37_-Pro_38_. Amino acid composition analysis of this fragment revealed six types of amino acids: Cys, Thr, Gly, Lys, Pro, and Ser. The ratios of Thr and Cys to Ser are approximately 2∶1. These data suggested that the C-terminal trypsin fragment was 8 amino acids in length and that Ser and Cys were the final two amino acids.

MS analysis of the C-terminal fragment yielded a mass of 1244 Da, which is larger by 238 Da than the calculated mass of the proposed eight amino acid (C-terminal amidated) peptide. This value is equivalent to the mass that would theoretically be added by O-linked palmitoylation of the Ser residue. Collectively, all our analytical data for δ/ω-PLTX-Pt1a were consistent with an amidated, 40 amino acid peptide structure ending with a palmitoyl Ser residue and a Cys residue.

### cDNA cloning

The correct order of the final two residues of δ/ω-PLTX-Pt1a was confirmed via cloned cDNA sequence coding for the δ/ω-PLTX-Pt1a precursor. δ/ω-PLTX-Pt1a cDNA was cloned via degenerate PCR techniques based on the experimentally determined sequence of the peptide. Degenerate primers from the interior of the toxin sequence were paired with vector specific primers to generate partial clones containing complete 5′ and 3′ end coding sequences. Perfectly matching primers overlapping each end sequence were then used to generate full-length cDNA. The sequence of the full-length cDNA coded for a 77 amino acid precursor of δ/ω-PLTX-Pt1a (Fig. 3B). The precursor contained a 20 amino acid consensus secretion signal separated from the coding sequence for the mature peptide by a 14 amino acid propeptide sequence similar to other toxin precursors in *Plectreurys* and other spiders [17], [27]. The coding region of the peptide sequence exactly matched our protein sequence data for the first 38 amino acids. It also confirmed our deduced composition for the final two residues and assigned their order as Ser_39_, Cys_40_ followed by a consensus C-terminal amidation sequence (Gly-Lys-Arg) that would be expected to yield Cys_40_ carboxyamide, consistent with predictions of our mass spectrometry data.

Based on these results, we propose that δ/ω-PLTX-Pt1a is a 40 amino acid peptide with an O-palmitoyl Ser_39_ and amidated C-terminal Cys_40_ (Fig. 3A). Calculated average mass for the proposed toxin with 5 disulfide bonds is 4646.4 Da. This value agrees with an experimentally determined mass of native δ/ω-PLTX-Pt1a of 4646 Da.

Figure 3C shows the comparison of complete amino acid sequences of δ/ω-PLTX-Pt1a, PLTX II and another insecticidal peptide Plt VI from *Plectreurys* venom reported by Quistad and Skinner [7]. All three peptides have very similar placement of the ten Cys residues, suggesting that the disulfide bridges may be conserved among these toxins. Interestingly, Plt VI also contains a C-terminal Ser residue. The reverse phase HPLC retention time for Plt VI suggests that it may also be palmitoylated.

### Effects of δ/ω-PLTX-Pt1a on synaptic transmission

**Figure 4:**
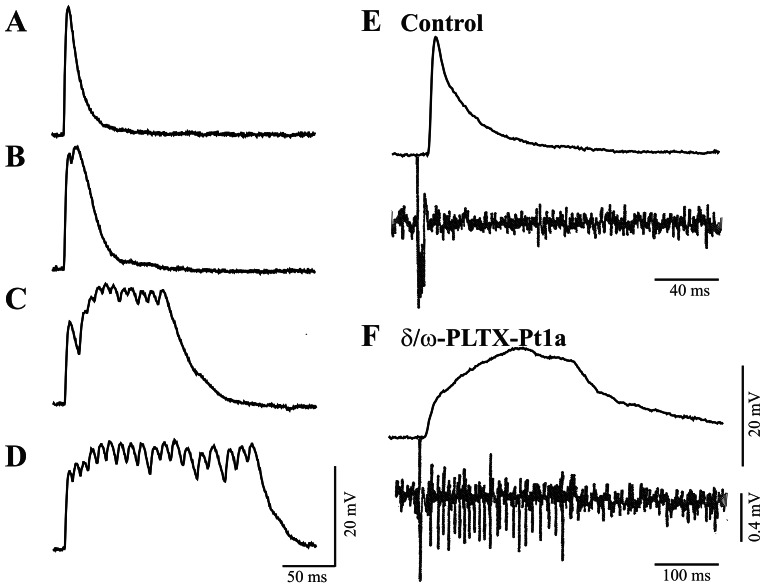
Excitatory effect of δ/ω-PLTX-Pt1a at *Drosophila* neuromuscular junctions. **A**. Control EJP recorded from the muscle of a third-instar larvae elicited by nerve stimulation. δ/ω-PLTX-Pt1a progressively prolonged stimulus evoked EJPs. Recordings were made 1 min (**B**), 3 min (**C**), and 5 min (**D**) after the application of 5 nM δ/ω-PLTX-Pt1a. **E**. Simultaneous intracellular recordings (top trace) from the larval muscle and extracellular recordings (bottom trace) from the motor nerve near its terminal region under control condition. **F**. In the presence of δ/ω-PLTX-Pt1a, a prolonged EJP is associated with a train of spikes originated at or near the nerve terminal. Representative figures from one of more than 20 independent experiments are shown.

The effect of δ/ω-PLTX-Pt1a on synaptic transmission was assayed on a *Drosophila* neuromuscular junction preparation. As shown in Figure 4, application of purified δ/ω-PLTX-Pt1a resulted in a significant prolongation of the excitatory junction potential (EJP) evoked by nerve stimulation. In the presence of 1–100 nM δ/ω-PLTX-Pt1a, the durations of prolonged EJPs ranged from several hundred milliseconds to several seconds, but the EJP amplitudes were not significantly reduced. The effect of δ/ω-PLTX-Pt1a was not easily reversible, as the prolonged EJPs persisted for more than 30 minutes after continuous washout with toxin-free saline (data not shown). On the other hand, δ/ω-PLTX-Pt1a did not induce spontaneous EJPs in the absence of nerve stimulation, nor did it cause a change in the membrane potential of the postsynaptic muscle cell. δ/ω-PLTX-Pt1a was also without effect on the amplitude (or frequency) of spontaneous, miniature EJPs (data not shown). These results are all consistent with a specific action of δ/ω-PLTX-Pt1a upon the electrical excitability of the presynaptic nerve terminal.

δ/ω-PLTX-Pt1a did induce a train of small spikes that were recordable with an extracellular suction electrode placed on the motor nerve close to the presynaptic terminal region (Fig. 4). Previously, we and others have shown that these repetitive motor axon spikes are likely initiated and sustained by the interaction between terminal Ca^2+^ spikes and axonal Na^+^ spikes, a positive feedback cycle that occurs at fly NMJs with enhanced presynaptic excitability [28]–[31]. As shown in Figure 4B, the time course of δ/ω-PLTX-Pt1a-induced axonal spikes corresponds to that of the prolonged EJPs, suggesting that it caused prolonged presynaptic excitation concomitant with augmented release of neurotransmitters.

### Effects of δ/ω-PLTX-Pt1a on neuronal ion channels

**Figure 5:**
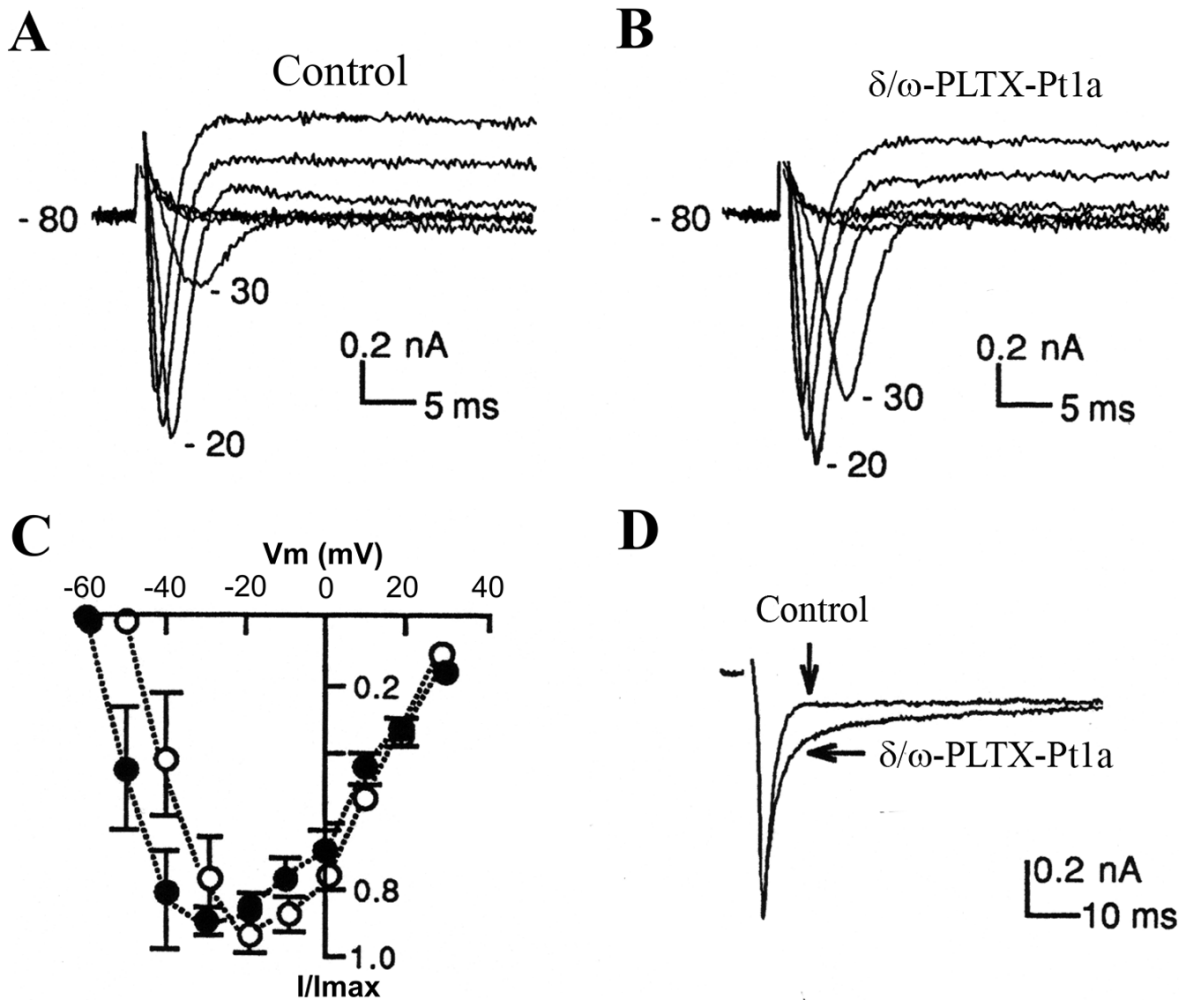
Effects of δ/ω-PLTX-Pt1a on voltage-dependent Na^+^ currents. Representative whole-cell Na^+^ currents recorded from a cultured *Drosophila* neuron, before (**A**) and after the application of 20 nM δ/ω-PLTX-Pt1a (**B**). Na^2+^ currents were evoked by a series of depolarizing voltage steps in 10 mV increments from a holding potential of −80 mV. (**C**) To compare the voltage-dependence of Na^+^ channel activation, peak Na^+^ currents before (open circles) and after δ/ω-PLTX-Pt1a (solid circles) were normalized to the maximum amplitude (I/Imax) and plotted against test potentials. (n = 7; mean ± S.E.). **D**. Representative Na^+^ current traces were normalized and aligned to show a slowing of channel inactivation by δ/ω-PLTX-Pt1a.

Membrane excitability of the presynaptic nerve terminal is precisely regulated by several classes of ion channels [28]–[31]. To directly examine the effect of δ/ω-PLTX-Pt1a on different neuronal ion channels, we recorded whole-cell currents in cultured *Drosophila* “giant” neurons using standard voltage clamp technique [22]. δ/ω-PLTX-Pt1a had no significant effect on voltage-gated K^+^ currents in *Drosophila* “giant” neurons (n = 5, data not shown). However, δ/ω-PLTX-Pt1a did affect Na^+^ currents in a couple of ways. Firstly, it shifted voltage-dependent activation of Na^+^ currents toward the hyperpolarizing direction (Figs. 5A-C). The threshold potential for Na^+^ channel activation was decreased about 7 mV by application of 20 nM δ/ω-PLTX-Pt1a (Table 1). In addition, we observed a slowing of Na^+^ channel inactivation kinetics by δ/ω-PLTX-Pt1a in one half of the neurons examined (Fig. 5D). On average, the time constant for decay of Na^+^ currents increased about 2-fold, while the kinetics of recovery from inactivation for Na^+^ currents was apparently not affected by δ/ω-PLTX-Pt1a (Table 1). Together, the above changes in Na^+^ channel properties would lead to neuronal hyperexcitability, i.e. Na^+^ activation at more negative potentials and the lengthened channel open time due to less complete inactivation following channel opening [32]–[34]. We observed that a stimulus of slightly lower intensity seemed to be required to generate action potentials in motor nerves after application of toxin.

**Table 1:** Effects of δ/ω-PLTX-Pt1a (20 nM) on the voltage-dependent Na^+^ currents.

	V_th_ (mV)^a^	V_h_ (mV)^b^	I_p_ (nA)^c^	I_ss_ (nA)^c^	I_p_/I_ss_	♦ (ms)^d^	r (%)^e^
**Before**	−38.6±9.0	−45.5±7.6	−1.43±0.64	−0.15±0.12	25.9±25.1	1.84±0.34	0.76±0.06
**After**	−45.7±9.7*	−50.0±5.4	−1.18±0.66	−0.22±0.15	9.2±9.4	3.44±1.58**	0.79±0.08
	n = 7	n = 4	n = 7	n = 7	n = 6	n = 14	n = 4

Results are shown as Mean±SD. * p<0.01. **p<0.002. (Paired student's t-test).

**a**. V_th_ is the threshold membrane potential at which the amplitude of inward Na^+^ current is larger than 50 pA which can be confidently distinguished from the noise level;

**b**. V_h_ is the membrane potential of half-inactivation;

**c**. I_p_ and I_ss_ are the amplitudes of peak and steady-state Na^+^ currents respectively;

**d**. ♦is the decay rate of the inactivating Na^+^ current evoked by depolarizing pulse to −20 mV from the holding potential of −80 mV. The value was obtained by single exponential fitting of the current decay phase. In 2 of the 14 cells, currents were better fitted with a double-exponential function, of which ♦_1_ was shown here.

**e**. r is the fractional recovery from inactivation of Na^+^ currents at −10 mV, assessed by the double pulse paradigm with an interpulse interval of 500 ms.

**Figure 6:**
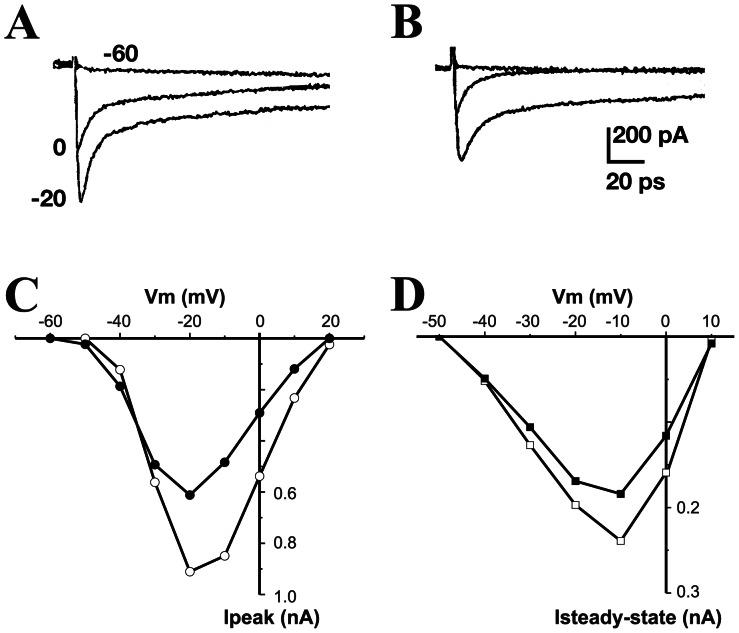
δ/ω-PLTX-Pt1a partially blocks neuronal voltage-dependent Ca^2+^ currents. Representative whole-cell Ca^2+^ currents recorded from a cultured *Drosophila* neuron, before (**A**) and after (**B**) the application of 100 nM δ/ω-PLTX-Pt1a. Ca^2+^ currents were evoked by a series of depolarizing voltage steps in 10 mV increments from a holding potential of −80 mV. For clarity, only three of the traces were shown. (**C**) and (**D**) show the I-V relations of peak and steady-state Ca^2+^ currents before (open symbols) and after addition of δ/ω-PLTX-Pt1a (solid symbols). Representative figures from one of five independent experiments are shown.

In view of the structure similarity between δ/ω-PLTX-Pt1a and the previously characterized Ca^2+^ channel blocker PLTX II (ω-PLTX-Pt1a), we also assessed a potential effect of δ/ω-PLTX-Pt1a on voltage-dependent Ca^2+^ channels. In these experiments, Ca^2+^ currents were isolated from Na^+^ and K^+^ currents by adding TTX to the bath solution and replacing K^+^ with Cs^+^ in the patch pipette following the established protocols for cultured *Drosophila* “giant” neurons [21], [22], [35], [36]. When activated by depolarizing voltage steps from a holding potential of −80 mV, most neurons exhibited both inactivating and non-inactivating Ca^2+^ currents (Fig. 6A). Application of δ/ω-PLTX-Pt1a partially inhibited both components of Ca^2+^ currents (Fig. 6B). As illustrated by the current-voltage relation (Figs. 6C & 6D), δ/ω-PLTX-Pt1a-induced block of Ca^2+^ currents was not dependent on the membrane potential.

**Figure 7:**
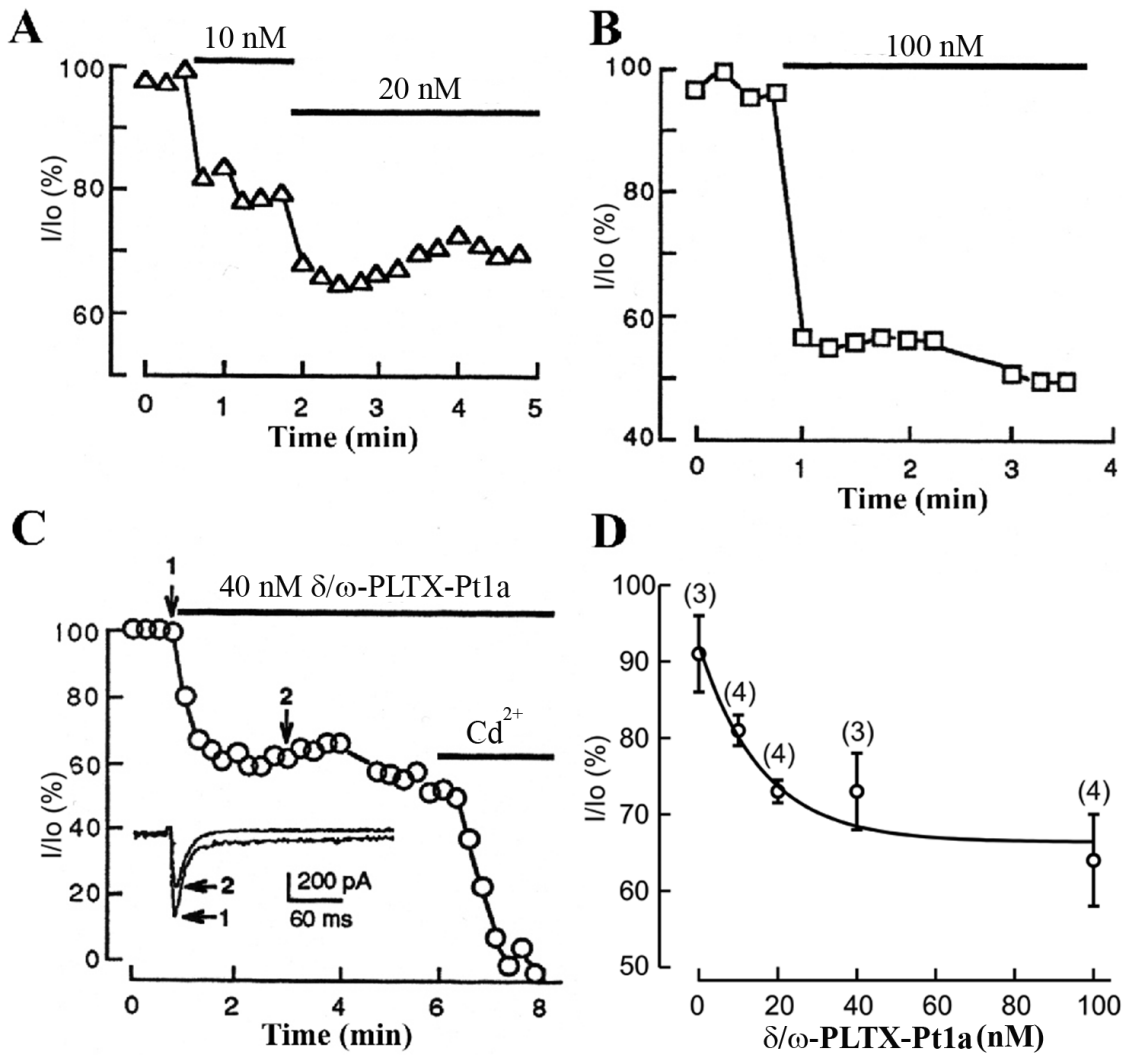
Time course and dose-dependence of δ/ω-PLTX-Pt1a's effect on Ca^2+^ currents. (**A**), (**B**) and (**C**) show the time course and extent of reduction in peak Ca^2+^ currents from three different neurons after additions of different concentrations of δ/ω-PLTX-Pt1a. In these experiments, neurons were repeatedly depolarized to −20 mV from a holding potential of −80 mV. The interpulse interval was 15 sec. Solid bars denote the duration of application of either δ/ω-PLTX-Pt1a or Cd^2+^. The insert in (**C**) shows current traces taken before and after 40 nM δ/ω-PLTX-Pt1a, as indicated by arrows 1 and 2. **D**. Dose-dependence of δ/ω-PLTX-Pt1a was determined as the percentage of peak Ca^2+^ currents in δ/ω-PLTX-Pt1a (I) relative to that of control (Io). The curve was fitted with a single exponent function. Values are shown as mean ± S.E.

To characterize the time course and dose-dependence of δ/ω-PLTX-Pt1a, Ca^2+^ currents were elicited by depolarizing pulse to −20 mV from a holding potential of −80 mV with an interpulse interval of 15 sec. δ/ω-PLTX-Pt1a at 10–100 nM concentrations rapidly reduced Ca^2+^ currents, as the plateau level was reached within 1 min after the application of the toxin (Fig. 7A-C). The remaining current was eliminated by Cd^2+^, indicating that δ/ω-PLTX-Pt1a acts only on a subset of the calcium channels present in this preparation (Fig. 7C). This result was further illustrated in the dose-response curve (Fig. 7D).

**Figure 8:**
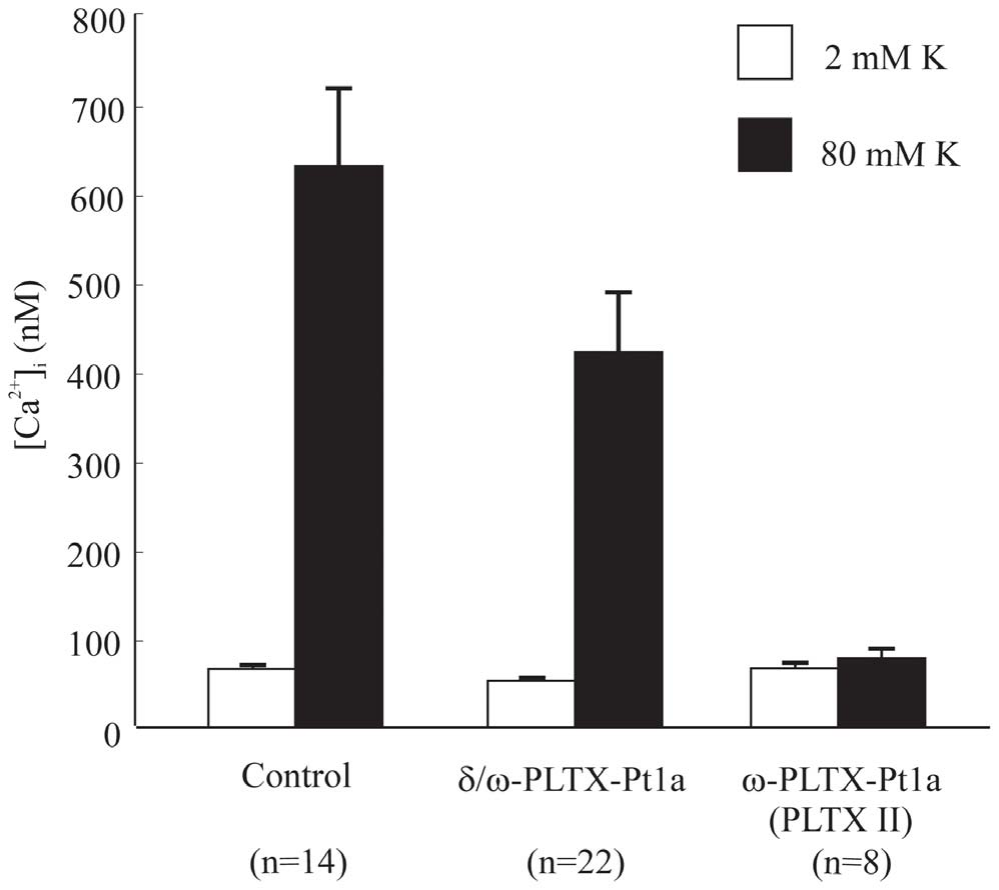
δ/ω-PLTX-Pt1a reduces stimulus-induced intracellular Ca^2+^ rise in cultured *Drosophila* neurons. Summary of average decreases in high K**^+^** induced intracellular Ca^2+^ rises produced by δ/ω-PLTX-Pt1a and ω-PLTX-Pt1a (PLTX II). 20 nM δ/ω-PLTX-Pt1a blocked a portion of stimulus-induced intracellular Ca^2+^ rises, while 5 nM ω-PLTX-Pt1a nearly blocked all the Ca^2+^ rises in cultured *Drosophila* embryonic neurons. Values are shown as mean ± S.E.

In addition, we verified the inhibitory effect of δ/ω-PLTX-Pt1a on neuronal Ca^2+^ channels using a Ca^2+^ imaging method. A brief intracellular [Ca^2+^] rise was elicited by briefly depolarizing cultured embryonic *Drosophila* neurons with increased external [K^+^]. Consistent with the whole-cell voltage clamp results (Figs. 6 & 7), δ/ω-PLTX-Pt1a produced a significant, but partial blockade of the stimulus-induced increase in intracellular [Ca^2+^] in this preparation (Fig. 8). PLTX II, which has been previously shown to block essentially all neuronal Ca^2+^ current in *Drosophila* was applied as a positive control and, indeed, eliminated virtually all of the increase in intracellular [Ca^2+^] (Fig. 8).

The sum of our physiology experiments indicates that δ/ω-PLTX-Pt1a is a functionally unique toxin that targets both Na^+^ and Ca^2+^ channels. On the one hand, δ/ω-PLTX-Pt1a enhanced the activity of neuronal Na^+^ channels by lowering voltage of activation and slowing inactivation, both of which would enhance neuronal excitability concomitant with prolonged EJPs at NMJs. On the other hand, since δ/ω-PLTX-Pt1a augmented synaptic transmission, the subset of neuronal Ca^2+^ channels blocked by δ/ω-PLTX-Pt1a should not be specifically involved in the release of neurotransmitters. It is however possible that a preferential inhibition of Ca^2+^ channel currents by δ/ω-PLTX-Pt1a may contribute to the increase in presynaptic excitability through indirectly decreasing Ca^2+^-activated K^+^ (K_Ca_) channels which are known to be functionally coupled to specific types of Ca^2+^ channels [37], [38].

## Discussion

### Structure of δ/ω-PLTX-Pt1a

We have identified and characterized δ/ω-PLTX-Pt1a, a palmitoylated peptide toxin, from the venom of the spider *Plectreurys tristis*. δ/ω-PLTX-Pt1a bears structural similarities to the potent, inhibitory Ca^2+^ channel blocker PLTX II that we previously characterized from the same venom. The two toxins are similar in size and share sequence homology, particularly in the number and placement of cysteine residues so that they are likely to have a similar disulfide bond structure (Fig. 3C). As with PLTX II [10], the structure of δ/ω-PLTX-Pt1a involves an O-linked palmitoylation in the immediate region of the C-terminus. δ/ω-PLTX-Pt1a is unique, however, in that the palmitoyl moiety is linked to δ/ω-PLTX-Pt1a at a Ser residue rather than a Thr residue.

Fatty acylation of proteins is a common post-translational modification that contributes to membrane tethering, protein sorting and other cellular processes [12], [13], [39]. For integral and membrane proteins, addition of palmitic acid has been recognized as one of the most frequent and versatile types of lipid modification [40]–[42]. Most palmitoylated proteins appear to be acylated at cysteine residues via a thioester bond. Though it is often accepted that proteins can also be acylated as oxyesters of Thr or Ser [43], [44], PLTX II was the first example of an O-palmitoylated peptide that was determined by thorough structural analysis and confirmed by complete chemical synthesis of the biologically active toxin [10], [11]. Our analysis of δ/ω-PLTX-Pt1a provides a clear example of O-palmitoyl Ser in a peptide and suggests that O-palmitoylation may be a general property for a group of structurally related peptide toxins in the venom of *Plectreurys tristis*. Interestingly, while spider-venom peptides are known to undergo post-translational modifications, palmitoylation has not been reported in any other spider venom [45]. The Plectreuridae comprise a small family of primitive hunting spiders found only in North America and Cuba. Only two living genera (*Plectreurys* and *Kibramoa*) are known [46]. O-palmitoylation of venom components may be an evolutionary specialization that is restricted to the Plectreuridae or to a subset of species within this family. However, given that the vast majority of spider-venom peptides are yet to be explored [18], [45], future studies may reveal similar palmitoylation in other spider species.

We have previously shown that fatty acylation is required for the biological activity of PLTX II. Similarly, we have not detected activity in deacylated δ/ω-PLTX-Pt1a. We have also seen a great loss of activity in complex mixtures of toxins from this venom, concomitant with a hydrophilic shift on HPLC, under conditions that would be expected to deacylate toxins with structures similar to PLTX II and δ/ω-PLTX-Pt1a. Further studies are necessary to elucidate the specific function of palmitoylation for these toxins. While highly specific and novel mechanisms are conceivable, it is possible that the lipid moiety may simply serve to tether the otherwise hydrophilic peptide to the plasma membrane by interacting with either the lipid bilayer or channel transmembrane regions. This might increase the probability of interaction of the peptide toxin with ion channels in the membrane or induce a conformational change in the target channel once the toxin is bound [47]. Similar mechanisms have been suggested for ω-agatoxin-Aa4a, which does not have the palmitoyl moiety, but does have a long hydrophobic C-terminal tail that is essential for its activity [2], [48]. Alternatively, palmitoylation might allow these plectotoxins to penetrate the cell membrane and act at an intracellular site on a channel or regulatory protein [49].

### Mechanism of δ/ω-PLTX-Pt1a effects on synaptic transmission

δ/ω-PLTX-Pt1a and the previously characterized PLTX II share some structural features and both toxins block neuronal Ca^2+^ channels. The two toxins, however, have very distinct effects on synaptic transmission at the *Drosophila* neuromuscular junction. While PLTX II potently and irreversibly inhibits synaptic transmission [5], [9], an immediate action of δ/ω-PLTX-Pt1a on synaptic transmission is excitatory. It prolongs the duration of EJPs evoked by stimulation of presynaptic nerves. Our data are consistent with the hypothesis that δ/ω-PLTX-Pt1a enhances synaptic transmission by increasing nerve terminal excitability and prolonging neurotransmitter release at the nerve terminals.

Hololena toxin (HoTX), a toxin purified from the spider *Holonela curta*, preferentially blocks a component of the Ca^2+^ current and blocks neurotransmitter release, indicating that in *Drosophila*, as in vertebrates, specific subtypes of channels are involved in transmitter release at specific nerve terminals [9], [50]. Given that δ/ω-PLTX-Pt1a enhances rather than inhibits synaptic transmission under normal conditions, the Ca^2+^ currents blocked by δ/ω-PLTX-Pt1a are apparently not required for neurotransmitter release, but may play a role in controlling the electrical properties of the nerve terminals. One possibility that requires further investigation is that the δ/ω-PLTX-Pt1a-sensitive Ca^2+^ channels are associated with K_Ca_ channels. In *Drosophila*, K_Ca_ channels are known to contribute significantly to the repolarization of action potentials, so that a reduction in their activation may prolong axonal terminal depolarization in response to an invading action potential and, concomitantly, prolong release of neurotransmitter, resulting in a prolonged EJP [30], [51], [52]. Previous studies in vertebrate neurons have also demonstrated that such functional coupling regulates neuronal excitability via specific subtypes of Ca^2+^ channels [37], [38]. Our findings suggest that a similar coupling may occur in *Drosophila*. In conjunction with Hololena toxin, δ/ω-PLTX-Pt1a may be useful in further characterization of specific subtypes of Ca^2+^ channels that are involved in either the release of neurotransmitters or control of excitability of nerve terminals in *Drosophila*.

We have also demonstrated excitatory activity of δ/ω-PLTX-Pt1a on neuronal Na^+^ channels. The excitatory effects on Na^+^ currents may lengthen the Na^+^ spike and help ensure that the action potential still invades the nerve terminal when a significant portion of the terminal Ca^2+^ current is blocked by the toxin. With a delayed inactivation, such altered Na^+^ spikes may act to facilitate further the prolonged depolarization of the terminal through positive feedback interaction with the remaining Ca^2+^ currents upon removal of the K_Ca_ repolarizing force [28], [30], [31].

The opposite but functionally synergistic actions of δ/ω-PLTX-Pt1a on Ca^2+^ and Na^+^ channels may lead to insights into the molecular basis of channel function, particularly if the binding sites for the toxin are similar in structure in both channel types. The dual action of the toxin may be similar to toxin cabals initially observed in the predatory cone snails [53]. Cabals are usually seen as groups of venom peptides that act synergistically to achieve the same physiological end point in targeted preys [8]. Our findings indicate that δ/ω-PLTX-Pt1a may have such cabal-like properties within a single toxin.

Based on our previous experience with (now commercially available) PLTX II [54], we expect δ/ω-PLTX-Pt1a to be chemically synthesizable in an active form. It may now also be possible to express active forms of well-characterized toxins from this venom in recombinant systems. The precursors of δ/ω-PLTX-Pt1a and similar toxins from the same venom are small and the biological activities of the toxins are dependent upon the presence of an O-palmitoyl moiety in the mature toxin, so it may be possible to ascertain the specific features of precursor structure that are required for O-palmitoylation to occur.
